# Mean peak systolic velocity of superior thyroid artery for the differential diagnosis of thyrotoxicosis: a diagnostic meta-analysis

**DOI:** 10.1186/s12902-019-0388-x

**Published:** 2019-06-06

**Authors:** Xiaojuan Peng, Shenglan Wu, Caiqun Bie, Huijun Tang, Zhe Xiong, Shaohui Tang

**Affiliations:** 1 Department of Gastroenterology, The First Affiliated Hospital, Jinan University, Guangzhou, 510630 Guangdong China; 2 Department of Endocrinology, Affiliated Hospital of Xiangnan University, Chenzhou, Hunan China; 3 Department of Gastroenterology, Shajing People’s Hospital of Bao’an Shenzhen, Guangdong, China

**Keywords:** Peak systolic velocity of superior thyroid artery, Thyrotoxicosis, Graves’ disease, Destructive thyrotoxicosis, Differential diagnosis, Diagnostic meta-analysis

## Abstract

**Background:**

Thyrotoxicosis is often caused by destructive thyroiditis (DT) or Graves’ disease (GD), and a prompt and accurate differential diagnosis for thyrotoxicosis is needed as management strategy differs. A meta-analysis of published literature was performed to evaluate the diagnostic accuracy for differentiating GD from DT patients by the measurement of mean peak systolic velocity of superior thyroid artery (STA-PSV) using ultrasonography.

**Methods:**

The databases of Embase, Pubmed, Cochrane, Web of Science, Wanfang, and CNKI were retrieved without time limit to identify eligible studies. The statistical information and scientific quality were assessed and classified. The data were analyzed using Stata12.0 software.

**Results:**

A total of 11 studies with 1052 cases only from Asia were included. Meta-analysis results showed the pooled sensitivity and pooled specificity of STA-PSV by ultrasonography were 0.86 (95% CI, 0.80–0.90) and 0.93 (95% CI, 0.86–0.97) in distinguishing GD from DT, respectively, with the AUC of 0.94 (95% CI, 0.92–0.96) .

**Conclusion:**

STA-PSV by ultrasonography is a useful diagnostic method in differentiating GD from DT. More studies from other countries are needed to further evaluate the accuracy of STA-PSV for the differential diagnosis of thyrotoxicosis.

## Background

Thyrotoxicosis refers to a condition associated with an increase of the levels of free triiodothyronine (FT3) and free thyroxine (FT4) in the blood circulation [1]. It can be classified into two categories: destruction-induced thyrotoxicosis and stimulation-induced thyrotoxicosis. Destruction-induced thyrotoxicosis is often seen in patients with destructive thyroiditis (DT), including postpartum thyroiditis, painless thyroiditis, amiodarone-induced thyrotoxicosis and subacute thyroiditis, whereas stimulation-induced thyrotoxicosis is often observed in Graves’ disease (GD) [2]. In terms of treatment, GD can be treated by antithyroidal medication, radiation therapy or thyroidectomy, but DT is generally treated conservatively [3]. Given that the different prognosis and treatments of these two conditions, it is extremely important to make a correct and rapid differential diagnosis from each other.

Radioactive iodine uptake (RAIU) has been recognized as the most accurate test for discriminating thyrotoxicosis [3], the sensitivity and specificity are 100 and 90%, respectively [4]. But the results can be affected by iodine-containing foods or drugs. What more, RAIU is contraindicated during gestation and lactation [5]. Serum thyroid stimulating hormone (TSH), ratio of serum tri-iodothyronine to thyroxine, serum thyroid stimulating hormone receptor antibodies (TRAb) and markers of inflammation also have been used to discriminate GD from DT [6].

The high intrathyroidal blood flow and increase in mean peak systolic velocity (PSV) of superior thyroid artery are signs of Graves’ disease [7]. Thyroid ultrasonography has enriched the diagnostic accuracy of thyroid diseases, including thyrotoxicosis [8]. The measurement of mean peak systolic velocity of superior thyroid artery (STA-PSV) by ultrasonography, which is easier and convenient, can provide qualitative and quantitative mention to clinicians in discriminating thyrotoxicosis [9]. However, no appropriate cut-off value criteria have been established about the STA-PSV, and the sensitivity and specificity of STA-PSV for the diagnosis of GD are different in different studies [10, 11]. In the present study, we performed a systematic literature review and meta-analysis to evaluate the diagnostic accuracy for differentiating GD from DT patients by STA-PSV.

## Methods

### Identification of studies

We searched the literatures published in Embase, Web of Science, PubMed, CNKI, Wanfang Data, and the VIP database before September 1, 2018. Key words searched were as follows: (“peak systolic velocity of superior thyroid artery”or“STA-PSV”or“color flow doppler sonography” or “doppler sonography” or “ultrasonography” or “echography” or “ultrasound”) and (“thyrotoxicosis” or “Graves’ disease” or “GD” or “destructive thyroiditis” or “DT” or “painless thyroiditis” or “postpartum thyroiditis” or “subacute thyroiditis”). Furthermore, other relevant published reports and the references of selected studies were also manually searched.

### Inclusion and exclusion criteria

Studies were included if they met the following criteria (1). The research types were diagnostic studies on the peak systolic velocity of superior thyroid artery by using ultrasonography in patients with Graves’ disease or destructive thyroiditis (2). Patients can be subdivided into two groups: those with destructive thyrotoxicosis and those with Graves’ disease group (3). Destructive thyrotoxicosis was diagnosed on the basis of the symptoms, T3 to T4 ratio less than 20, T3 and T4 concentrations increased and TSH concentration decreased lasting for fewer than 3 months and/or later development of hypothyroidism, and/or low uptake on pertechnetate thyroid scan. Graves’ disease was diagnosed on the basis of clinical parameters, eye signs, T3 to T4 ratio greater than 20, and increased uptake on pertechnetate thyroid scan (4). The sensitivity and specificity (the number of true-positive, true-negative, false-positive and false-negative results) and their corresponding 95% confidence intervals (CIs), were provided or can be calculated. Abstracts, reviews, case reports, repeated publications were excluded.

### Study selection and data extraction

After reading the title and abstract, the first selection was carried out by Xiaojuan Peng. Then, the full paper of qualified study was obtained. Xiaojuan Peng and Shenglan Wu assessed qualified studies for inclusion independently. Different opinions were resolved by discussing and consulting Shaohui Tang. The following were extracted from each selected study: first author name, country of study, year of publication, number of patients with thyrotoxicosis, destructive thyroiditis and Graves’ disease, cut-off value, raw data for analyzing sensitivity and specificity (the number of true-positive, true-negative, false-positive and false-negative) from the included studies.

### Assessment of methodological quality

QUADAS-2 (Quality Assessment of Diagnostic Accuracy Studies-2) was used to assess the quality of the included studies. The QUADAS-2 form is consists of four domains: (1) patient selection, (2) index test, (3) reference standard, and (4) flow and timing. Each domain is assessed in terms of risk of bias, and the first 3 domains are also assessed in terms of concerns about applicability. Signaling questions are included to help judge risk of bias. Risk of bias is judged as “low”, “high” or “unclear”. If the answers to all signaling questions for a domain are “yes”, then risk of bias can be judged low. If any signaling question is answered “no”, potential for bias exists. The “unclear” category should be used only when date are insufficiently reported to permit a judgment. Applicability sections are structured in a way similar to that of bias sections but do not include signaling questions. Concerns about applicability are rated as “high”, “low” or “unclear”. The results of quality assessment were used to provide an evaluation of the overall quality of included studies and to investigate potential sources of heterogeneity [12].

### Statistical analysis

STATA version 12.0 (Stata Corp, College Station, Texas) was used to perform the statistical analysis. Statistical heterogeneity between studies was examined using the I^2^ value. If the heterogeneity was acceptable (I^2^ < 50%), a fixed-effects model was used; conversely, random-effects model was used. In this study, the following data were calculated: threshold effect, spearman correlation coefficient, diagnostic odds ratio (DOR, used to eliminate possible threshold effect), sensitivity, specificity, positive likelihood ratio (PLR), negative likelihood ratio (NLR) and area under receiver operating characteristic curve (AUC) .

## Results

### Search results and study characteristics

**Fig. 1:**
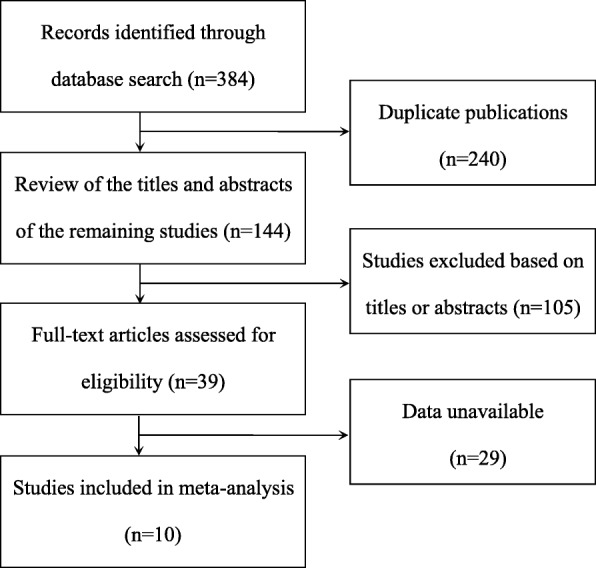
Flowchart of the study selection strategy

384 relevant articles were identified in the initial search, and 240 were duplicates. In the remaining articles, after the titles and abstracts were reviewed, 104 articles were excluded. Full-text articles of the remained 40 articles were reviewed. Of these, 30 articles were not diagnostic studies, so they were excluded. Finally, 10 published articles [1, 5, 6, 10–17] were included. The process of study selection was summarized in Fig. 1. The 11 studies included 1052 patients, namely, 706 patients with Graves’ disease and 346 patients with destructive thyroiditis. In all patients, their thyroid function assessment by measurement of TSH, total T3, and free T4 were performed, and color-flow doppler ultrasonography of the thyroid gland were done. Population and characteristics of the included studies were listed in Table 1, and the results of ultrasonography of the study participants were listed in Table 2.

**Table 1:** Population and characteristics of included studies

study	Country	No. of GD/ DT	Sex (male/female)		Age(year)	
			GD	DT	GD	DT
Kumar, 2009	India	34/31	10/24	4/27	37.1 (12.7)	29.1 (9.2)
Kim, 2015	Korea	40/20	12/28	7/13	43.23 ± 14.56	41.7 ± 13.97
Uchida, 2010	Japan	44/13	15/29	3/10	42.1 ± 14.4	43.8 ± 17.0
Zhao, 2012(1)	China	103/32	29/74	12/20	45.86 ± 13.6	43.06 ± 12.3
Zhao, 2012(2)	China	118/51	42/76	18/33	39.66 ± 13.7	45.06 ± 12.1
Hiraiwa, 2013	Japan	68/33	15/53	6/27	41 (33–54.5)	37 (29–43)
Karakas, 2014	Turkey	17/7	11/6	6/1	44.3 ± 17	43.7 ± 14.6
Shafi, 2012	Turkey	31/41	0/31	0/41	29.1 ± 5.12	29.1 ± 4.8
Xue, 2015	China	35/33	0/35	0/33	27.25 ± 4.00	26.88 ± 4.03
Yang, 2017	China	30/25	0/30	0/25	not clear	not clear
Donkol, 2013	Saudi Arabia	18/8	not clear	not clear	31.1 ± 8.4	33.1 ± 7.5

*GD* Graves’ disease, *DT* destructive thyroiditis, (1) retrospective study, (2) prospective study

**Table 2:** The result of ultrasonography of the study participants

study	linear transducer	US equipment	STA-PSV (cm/s)		Cut-off (cm/s)	Sensitivity(%)	Specificity(%)
			GD	DT			
Kumar, 2009	7.5-MHz	Philips, USA	57.6 ± 13.1	22.4 ± 5.4	40	94	100
Kim, 2015	5–12-MHz	Phillips, USA	78.96 ± 29.04	29.97 ± 14.67	41.3	95	85
Uchida, 2010	10-MHz	Hitachi, Japan	78.48 ± 36.28	28 ± 12.84	45	89	92
Zhao, 2012(1)	7.5-MHZ	Aloka A10	70.16 ± 32.6	38.86 ± 21.5	50.5	71	97
Zhao, 2012(2)	7.5-MHZ	Aloka A10	67.93(56.0–95.7)	33(27–40)	50.5	81	96
Hiraiwa, 2013	7.5–8 MHz	Tochigi, Japan	58.9 (49.3–79.5)	25.6 (17.4–33.9)	43	87	100
Karakas, 2014	5–12-MHz	Bothell, USA	138 (107–301)	54 (37–59)	87	100	100
Shafi, 2012	7–14-MHz	Toshiba, Japan	37.58 ± 10.89	24.3 ± 7.3	29.4	77	78
Xue, 2015	N/A	Logiq E9, USA	62.143 ± 13.772	38.423 ± 11.742	40	83	82
Yang, 2017	10-MHz	Phillips IE33	66.79 ± 9.19	45.70 ± 7.96	60	80	88
Donkol, 2013	3–9 MHz	Phillips, USA	50.4 ± 23.4	21.7 ± 14.8	40	89	88

*GD* Graves’ disease, *DT* destructive thyroiditis, *STA-PSV* peak systolic velocity of superior thyroid artery, *US equipment* ultrasonography equipment, (1) retrospective study, (2) prospective study

### Study quality

**Fig. 2:**
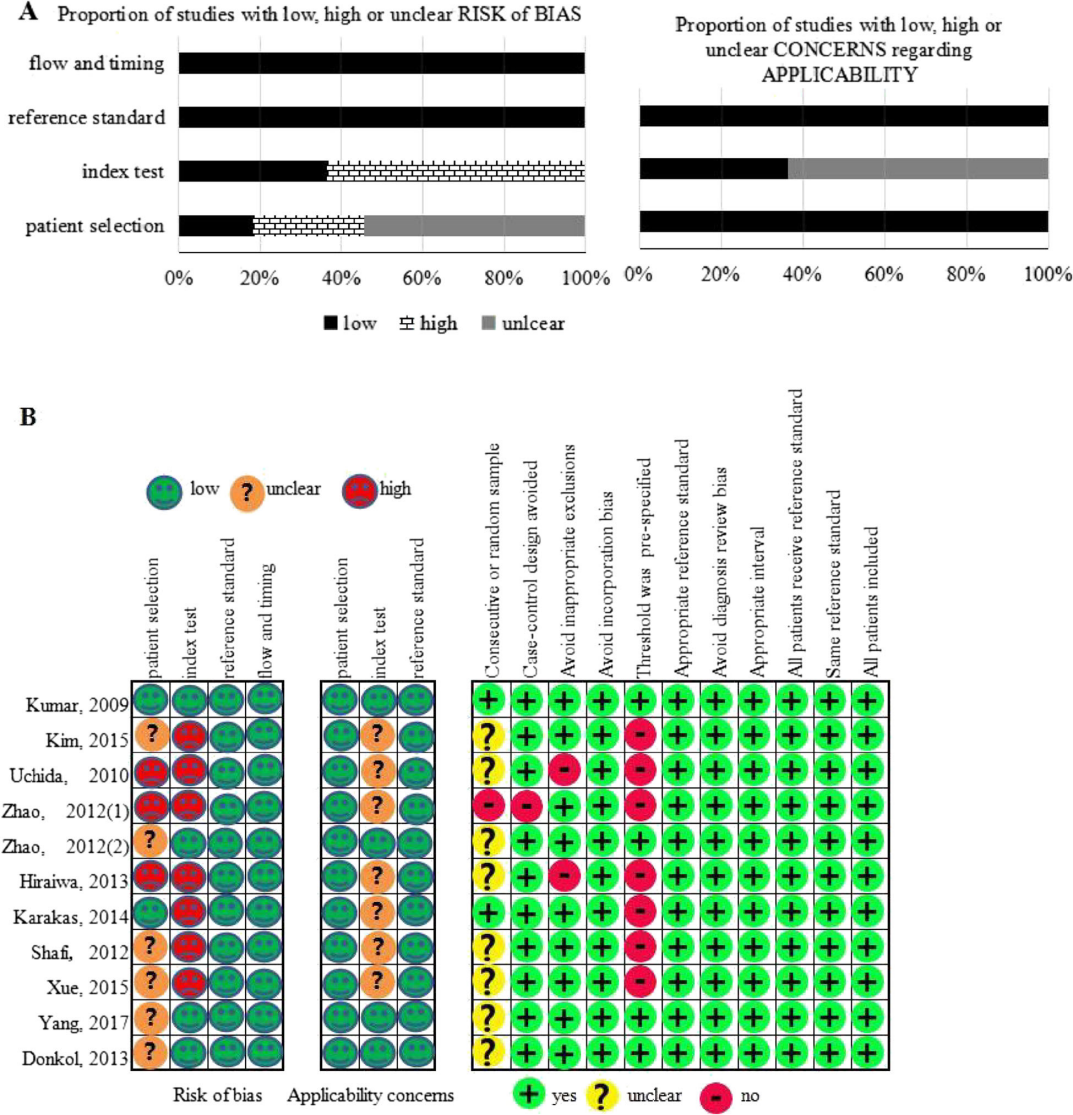
Methodological evaluation according to QUADAS-2 of the included studies **a** overall and **b** by study

According to QUADAS-2, the result of the evaluation of the risk of bias and concerns regarding applicability of the included studies was reported in Fig. 2 a, b. As shown in Fig. 2, the included studies were generally of high quality.

### Combined results

**Fig. 3:**
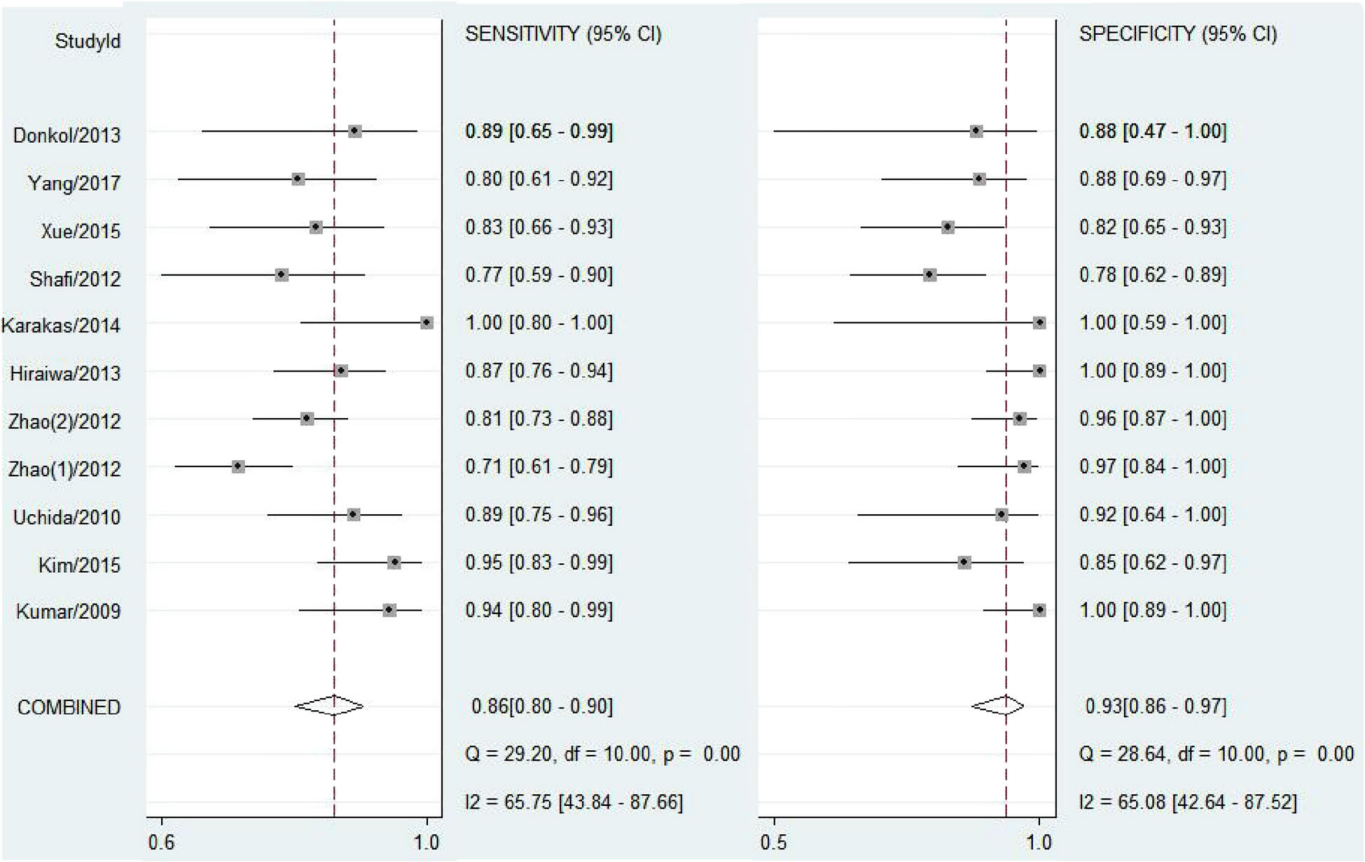
Forest plots of sensitivity and specificity of STA-PSV in distinguishing GD from DT. Plots display diagnostic probabilities of included studies, corresponding 95% confidence intervals

**Fig. 4:**
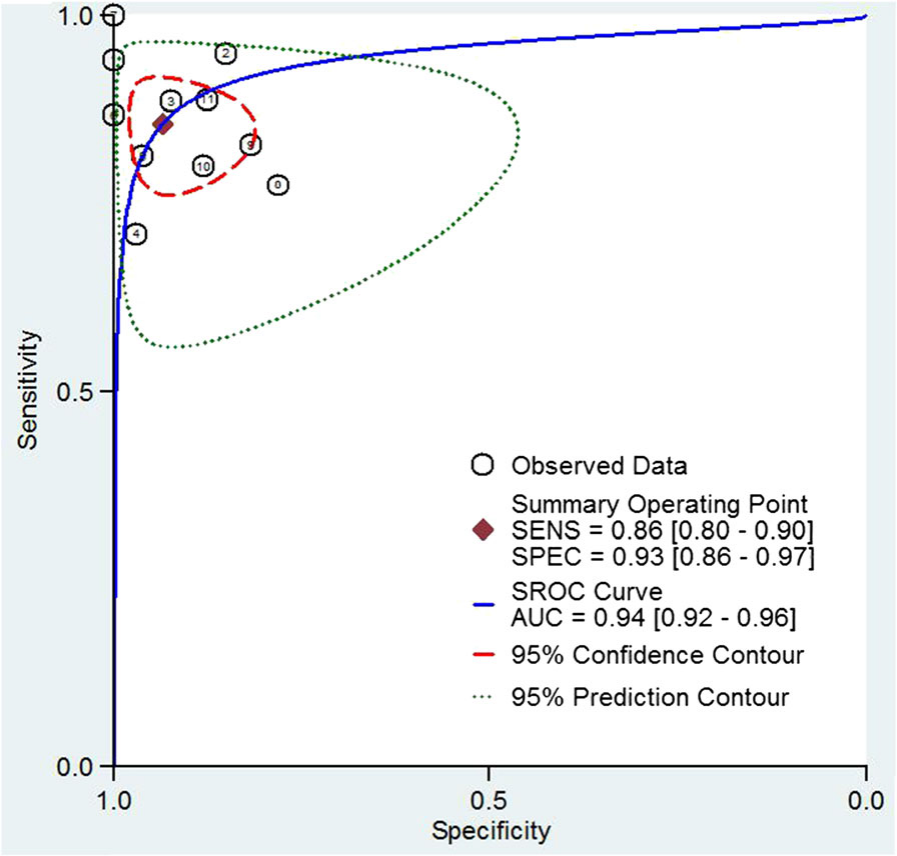
Receiver operating characteristic graph of STA-PSV in distinguishing GD from DT, with 95% confidence region and 95% prediction region. Individual study estimates are represented as circles

The test for the heterogeneity among the studies showed significant heterogeneity (I^2^ = 65.75 and 65.08% for sensitivity and specificity, respectively), so the random-effects model was used. Meta-analysis results showed the pooled sensitivity and pooled specificity of STA-PSV by ultrasonography were 0.86 (95% CI, 0.80–0.90) and 0.93 (95% CI, 0.86–0.97) in distinguishing GD from DT, respectively (Fig. 3), with the area under receiver operating characteristic curve (AUC) of 0.94 (95% CI, 0.92–0.96) (Fig. 4), which was similar to the diagnostic accuracy for GD by radioactive iodine uptake (sensitivity 100%, specificity 90%) [4]. The average likelihood ratio of the positive and negative test result was calculated on the basis of the pooled estimates of sensitivity and specificity, and the results showed PLR and NLR of STA-PSV in differentiating GD from DT patients were 13.0 (95% CI, 6.1–27.8) and 0.15 (95% CI, 0.10–0.22), respectively. In addition, the mean DOR value of STA-PSV was 85 (95% CI, 33–220). In later steps of this meta-analysis, we repeated calculating all these pooled sensitivity and specificity with each of the 11 studies removed individually, and found that the final results were very nearly the same as the initial result. These findings reflect the stability and credibility of the results of this meta-analysis. For publication bias analysis, Deeks funnel plot asymmetry test was not significant (*p* = 0.97).

## Discussion

It is difficult to differentiate subclinical or mild GD from DT because of the absence of specific signs, such as ophthalmopathy, skin and nail changes. RAIU is the gold standard, but high cost, limited availability and contraindications to a radioisotope scan during pregnancy and lactation may restrict its application [15]. TSH receptor antibody level also can help in aetiological differentiation of thyrotoxicosis in difficult situations [18], while TSH-receptor-stimulating immunoglobulin bioassays are also costly and time-consuming. Color flow doppler ultrasonography (CFDS), a cost-effective, portable, safe, and noninvasive method [8], is now widely used to measure tissue vascularization and blood flow. CFDS of the thyroid gland, both qualitative and quantitative [19], helps in assessing thyroid gland functional status indirectly by studying the vascularity [7].

Compared with thyroid CFDS judgment, STA-PSV detection is more objective and accurate [20]. One study [19] reported that STA-PSV > 40 cm/s had a sensitivity of 94.0% and a specificity of 100.0% for the differential diagnosis of GD and DT in 65 patients with thyrotoxicosis. However, another study [10] showed the sensitivity and specificity of STA-PSV > 50.5 cm/s in distinguishing GD from DT in 304 patients with thyrotoxicosis were 70.87 and 96.88%, respectively.

It was reported that Graves’disease accounted for 95% of the cases with hyperthyroidism during pregnancy [21]. Poorly controlled Graves’ disease during pregnancy can cause serious complications in both the mother and the fetus [22], such as low birth weight [23], preterm birth [24], and congenital malformations [25]. Therefore, early diagnosis is essential to successful management. Because of many of the signs and symptoms are similar to normal physiologic changes that occur in pregnancy, diagnosing hyperthyroidism during pregnancy is challenging [26]. Ultrasonography may be a good choice for pregnancy, not only because of its relatively low cost, real-time capability, safety, and operator comfort and experience, but also due to the security and free of radioaction [27]. It was reported that the sensitivity of STA-PSV in differentiating GD from hyperthyroidism in pregnancy was 80–83% [14, 16].

In the present meta-analysis including 11 studies, we evaluated the accuracy of STA-PSV for the differential diagnosis of GD and DT, which is the first meta-analysis reported in this field. Our results showed that the pooled sensitivity and specificity of STA-PSV in differentiating GD from DT were 0.86 (95% CI, 0.80–0.90) and 0.93 (95% CI, 0.86–0.97), respectively, and the AUC was 0.94 (95% CI, 0.92–0.96). The AUC value ranges between 0 and 1, higher value indicating better test performance. Furthermore, the pooled PLR and NLR of STA-PSV in differentiating GD from DT were 13.0 (95% CI, 6.1–27.8) and 0.15 (95% CI, 0.10–0.22), respectively, and the mean DOR value of STA-PSV in differentiating GD from DT was 85 (95% CI, 33–220). Likelihood ratios greater than 10 or less than 0.1 generate large and often conclusive changes from pre-test to post-test probability, likelihood ratios of 5 to 10 and 0.1 to 0.2 generate moderate shifts in pre-test to post-test probability [28], and higher DOR value indicates higher accuracy. Taken together, the results indicate that STA-PSV by ultrasonography has a better diagnostic accuracy in the differentiation of GD from DT.

There were some limitations to this meta-analysis. Firstly, this meta-analysis only included 11 studies and 1052 subjects, so further subgroup analysis could not be performed due to a small number of included diagnostic studies and patients. Secondly, this meta-analysis coverage in the world was limited because that all included studies were from Asia, and there was no study from other area. Therefore, the value of our results is limited for other areas except for the countries involved in the study. Lastly, threshold effect and other potential factors might have influenced the results, although the sensitivity analysis was performed and reflected the stability and credibility of the results of this meta-analysis.

## Conclusions

STA-PSV by ultrasonography is a useful diagnostic method in differentiating GD from DT. More studies from other countries are needed to further evaluate the accuracy of STA-PSV for the differential diagnosis of thyrotoxicosis.

## Data Availability

No additional data or materials are available.

## References

[1] Karakas O, Karakas E, Cullu N (2014). An evaluation of thyrotoxic autoimmune thyroiditis patients with triplex doppler ultrasonography. Clin Imaging.

[2] Ota H, Amino N, Morita S (2007). Quantitative measurement of thyroid blood flow for differentiation of painless thyroiditis from graves’ disease. Clin Endocrinol (Oxf).

[3] Bahn Chair RS, Burch HB, Cooper DS (2011). Hyperthyroidism and other causes of thyrotoxicosis: management guidelines of the American Thyroid Association and American Association of Clinical Endocrinologists. Thyroid.

[4] Kurita S, Sakurai M, Kita Y (2005). Measurement of thyroid blood flow area is useful for diagnosing the cause of thyrotoxicosis. Thyroid.

[5] Hari Kumar KV, Pasupuleti V, Jayaraman M (2009). Role of thyroid doppler in differential diagnosis of thyrotoxicosis. Endocr Pract.

[6] Uchida T, Takeno K, Goto M (2010). Superior thyroid artery mean peak systolic velocity for the diagnosis of thyrotoxicosis in Japanese patients. Endocr J.

[7] Castagnone D, Rivolta R, Rescalli S (1996). Color Doppler sonography in Graves' disease: value in assessing activity of disease and predicting outcome. AJR Am J Roentgenol.

[8] Alzahrani AS, Ceresini G, Aldasouqi SA (2012). Role of ultrasonography in the differential diagnosis of thyrotoxicosis: a noninvasive, cost-effective, and widely available but underutilized diagnostic tool. Endocr Pract.

[9] Kahaly GJ, Bartalena L, Hegedüs L (2018). 2018 European thyroid association guideline for the Management of Graves’ hyperthyroidism. Eur Thyroid J.

[10] Zhao X, Chen L, Li L (2012). Peak systolic velocity of superior thyroid artery for the differential diagnosis of thyrotoxicosis. PLoS One.

[11] Zuhur SS, Ozel A, Velet S (2012). Is the measurement of inferior thyroid artery blood flow velocity by color-flow Doppler ultrasonography useful for differential diagnosis between gestational transient thyrotoxicosis and graves’ disease? A prospective study. Clinics.

[12] Whiting PF, Rutjes AW, Westwood ME (2011). QUADAS-2: a revised tool for the quality assessment of diagnostic accuracy studies. Ann Intern Med.

[13] Kim TK, Lee EJ (2015). The value of the mean peak systolic velocity of the superior thyroidal artery in the differential diagnosis of thyrotoxicosis. Ultrasonography.

[14] Hiraiwa T, Tsujimoto N, Tanimoto K (2013). Use of color doppler ultrasonography to measure thyroid blood flow and differentiate graves’ disease from painless thyroiditis. Eur Thyroid J.

[15] Xue M, Shi QL, Tan KN (2016). The role of color doppler ultrasonography, thyroid function and auto antibody for the screening of Graves' disease in pregnancy. Zhonghua Nei Ke Za Zhi.

[16] Donkol RH, Nada AM, Boughattas S (2013). Role of color Doppler in differentiation of Graves' disease and thyroiditis in thyrotoxicosis. World J Radiol.

[17] Yang SG, Lin SM (2017). Value of peak blood flow velocity of superior thyroid artery in the identification of hyperthyroidism in pregnancy and pregnancy with graves disease. China Med Pharmacy.

[18] Izumi Y, Hidaka Y, Tada H (2002). Simple and practical parameters for differentiation between destruction induced thyrotoxicosis and graves’ thyrotoxicosis. Clin Endocrinol.

[19] Kumar KV, Vamsikrishna P, Verma A (2009). Utility of colour Doppler sonography in patients with graves’ disease. West Indian Med J.

[20] Erdoğan MF, Anil C, Cesur M (2007). Color flow Doppler sonography for the etiologic diagnosis of hyperthyroidism. Thyroid.

[21] Burrow GN (1985). The management of thyrotoxicosis in pregnancy. N Engl J Med.

[22] Yoshihara A, Noh J, Yamaguchi T (2012). Treatment of graves’ disease with antithyroid drugs in the first trimester of pregnancy and the prevalence of congenital malformation. J Clin Endocrinol Metab.

[23] Phoojaroenchanachai M, Sriussadaporn S, Peerapatdit T (2001). Effect of maternal hyperthyroidism during late pregnancy on the risk of neonatal low birth weight. Clin Endocrinol.

[24] Luewan S, Chakkabut P, Tongsong T (2011). Outcomes of pregnancy complicated with hyperthyroidism: a cohort study. Arch Gynecol Obstet.

[25] Galofre JC, Davies TF (2009). Autoimmune thyroid disease in pregnancy: a review. J Women's Health (Larchmt).

[26] Patil-Sisodia K, Mestman JH (2010). Graves hyperthyroidism and pregnancy: a clinical update. Endocr Pract.

[27] Reddy UM, Filly RA, Copel JA (2008). Prenatal imaging: ultrasonography and magnetic resonance imaging. Obstet Gynecol.

[28] Jacschke R, Guyart GH, Sackett DL (1994). User 's guides to the medical literature. III. How to use an article about a diagnostic test. B. What are the results and will they help me in earing for my patients? The evidence-based medicine working group. JAMA.

